# Myocarditis and heart function impairment occur in neonatal mice following in utero exposure to the Zika virus

**DOI:** 10.1111/jcmm.16064

**Published:** 2021-02-15

**Authors:** Chongzhi Bai, Jianqing Hao, Shihua Li, George Fu Gao, Yu Nie, Pengcheng Han

**Affiliations:** ^1^ Central Laboratory Shanxi Province Hospital of Traditional Chinese Medicine Taiyuan China; ^2^ CAS Key Laboratory of Pathogenic Microbiology & Immunology Institute of Microbiology Chinese Academy of Sciences Beijing China; ^3^ Shanxi Academy of Advanced Research and Innovation Taiyuan China; ^4^ School of Basic Medical Sciences Shanxi Medical University Taiyuan China; ^5^ State Key Laboratory of Cardiovascular Disease National Center for Cardiovascular Disease Chinese Academy of Medical Sciences and Peking Union Medical College Fuwai Hospital Beijing China; ^6^ Department of Biomedical Engineering Emory University Atlanta GA USA

Human cardiovascular diseases can be caused by infection with various viruses, such as coxsackievirus, influenza virus, human herpesvirus, Epstein‐Barr virus, hepatitis C virus, human immunodeficiency virus^1^ or severe acute respiratory syndrome coronavirus 2,^2^ which is currently circulating worldwide. It has been reported that infection with Zika virus (ZIKV) leads to severe neurological disorders,^3^ testis damage,^4^ and heart failure and arrhythmias.^5^, ^6^ A hypothesis for heart defects associated with intrauterine exposure to ZIKV was proposed by Angelidou et al^7^ who reported a case of congenital heart disease in one infant with congenital Zika syndrome. In addition, three other studies reported that antenatal exposure to ZIKV was related to cardiac defects in infants,^8^, ^9^, ^10^ but the pathophysiological mechanisms by which ZIKV leads to impairment of heart function are still unclear.

Here, we established an in utero transmission model of ZIKV infection by injecting 10^4^ plaque‐forming units (pfu) of ZIKV into the embryonic placenta of 2‐weeks pregnant BALB/c mice. Among the 11 newborns, three neonatal mice presented paralytic symptoms, implying severe neurological disorders. We performed immunohistochemistry with monoclonal antibody Z6^4^ to determine whether ZIKV direct infection occurred both in the neonatal brain and heart at day 9 after birth. High levels of ZIKV were detected in both the heart and brain of paralytic mice (Figure 1A, infected). The other eight newborn mice exhibited normal phenotype, and no virus was detected (Figure 1A, uninfected). To examine whether direct infection occurred in the myocardium of neonatal mice, ZIKV was detected by immunofluorescence using the monoclonal antibody Z6. We found that Z6 was localized in cardiomyocytes (α‐actinin^+^) of ZIKV‐infected mice 9 days after birth, and Z6 was not observed in the hearts of uninfected mice (Figure 1B). Echocardiography demonstrated left ventricle dilation and deterioration of fractional shortening and ejection fraction at days 4 and 9 after birth in ZIKV‐infected mice compared to uninfected mice and mock group (Figure 1C‐F). Electrochemiluminescence assays revealed that the level of serum muscle/brain creatine kinase (CK‐MB) and troponin T (cTnT) was dramatically elevated at days 4 and 9 after birth in ZIKV‐infected mice compared to uninfected mice (Figure 1G,H), indicating that cardiac injury occurred. Our results indicated that foetal hearts are susceptible to ZIKV infection and that ZIKV can directly infect the myocardium and lead to heart dysfunction in ZIKV‐infected neonatal mice.

**Figure 1:**
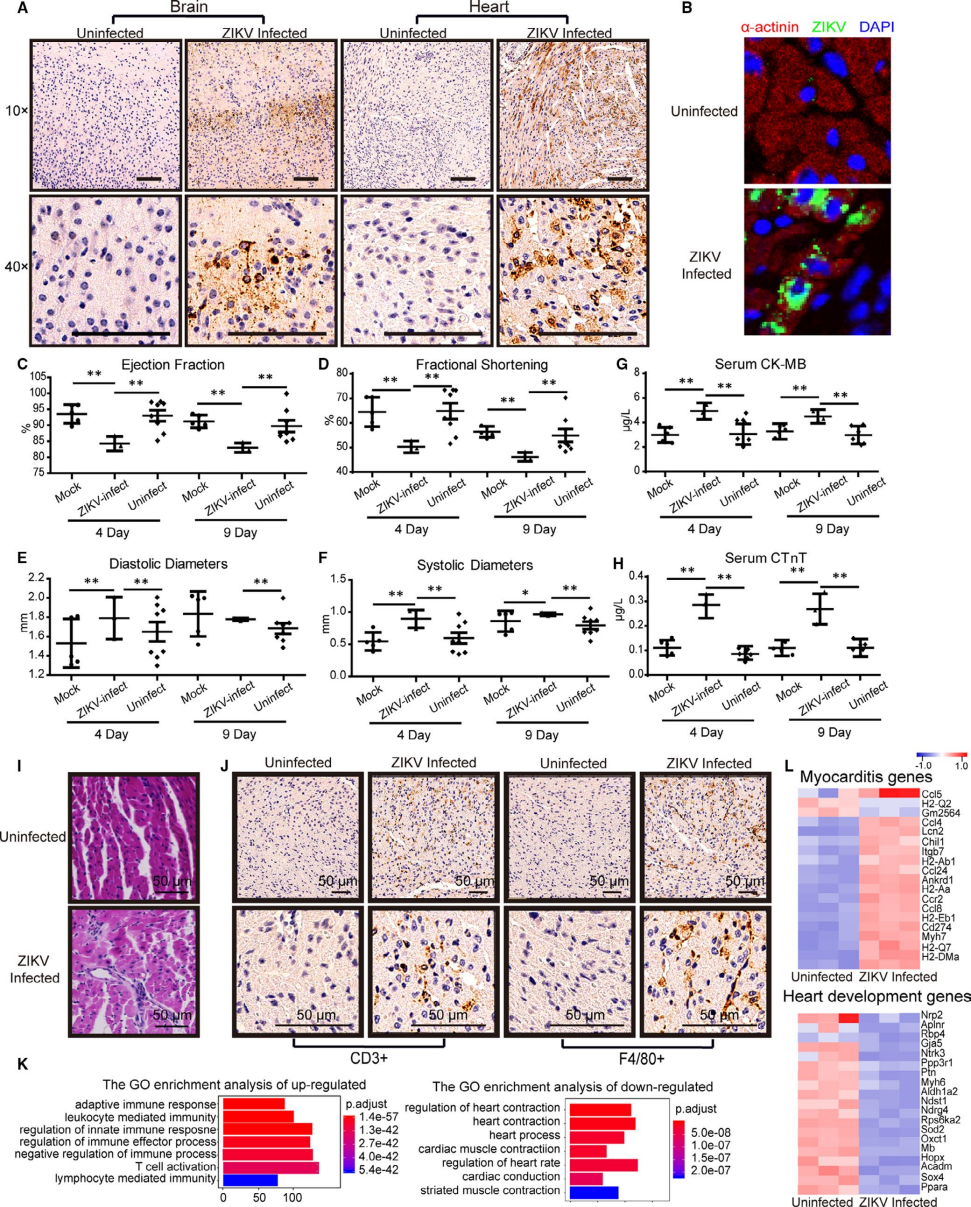
In utero exposure of foetal mice to ZIKV leads to myocarditis and heart function impairment. A, Immunohistochemistry of heart tissue sections stained for ZIKV in the brain and heart of offspring at day 9 after birth. In the infected and uninfected groups, n = 3 and n = 8 mice were included, respectively. Representative results from one mouse are displayed. B, Immunostaining shows that ZIKV (green) is detectable in cardiomyocytes (α‐actinin^+^, red) of ZIKV‐infected mice 9 d after birth. C‐F, Echocardiography revealed that ejection fraction and fractional shortening were decreased at days 4 and 9 after birth (C, D), and diastolic and systolic diameters increased at days 4 and 9 after birth (E, F). Mock (n = 5), ZIKV‐infected (n = 3) and uninfected (n = 8) offspring, respectively. Each dot represents one mouse. *P < .05; **P < .01. G, H, ECLA shows that serum CK‐MB and cTnT levels are elevated in ZIKV‐infected mice at days 4 and 9 after birth. Mock (n = 5), ZIKV‐infected (n = 3) and uninfected (n = 8) offspring, respectively. Each dot represents one mouse. *P < .05; **P < .01. I, J, Haematoxylin‐eosin staining displays monocyte infiltration in the myocardium. Immunohistochemistry showed that the numbers of F4/80^+^ macrophages and CD3^+^ leucocytes were significantly increased in the myocardium of ZIKV‐infected mice compared to uninfected mice at 9 d after birth. K, Gene ontology (GO) analysis of up‐regulated (left) and down‐regulated genes (right), respectively, based on RNA‐Seq data. The top 10 GO terms are shown. L, Heat maps showing hierarchical clustering of enriched differentially expressed genes between ZIKV‐infected and uninfected mouse hearts. Each lane represents the result from one mouse. n = 3 in each group

ZIKV is associated with myocarditis and heart failure in patients.^6^ We performed haematoxylin‐eosin staining of neonatal mouse heart sections and found that ZIKV causes massive monocyte infiltration in the myocardium at 9 days after birth (Figure 1I). Immunohistochemistry showed that the numbers of F4/80^+^ macrophages and CD3^+^ leucocytes were significantly increased in the myocardium at day 9 after birth in ZIKV‐infected mice compared to uninfected mice (Figure 1J). Gene ontology analysis revealed that up‐regulated genes were enriched for immune‐related pathways, whereas down‐regulated genes were enriched for pathways related to heart function (Figure 1K). Simultaneously, the levels of myocarditis‐related genes, including *Ccl5* ^11^ and *Myh7*,^12^ are increased, whereas the expression of heart development‐related genes, including *Nrp2* ^13^ and *Rbp4*,^14^ was decreased in heart tissue as demonstrated by RNA sequencing (Figure 1L).

Together, we established an in utero transmission model of ZIKV infection by placenta injection in 2‐weeks pregnant BALB/c mice, based on the contemporary strain ZIKV‐SMGC‐1. These data indicate that ZIKV can directly infect cardiomyocytes, causing myocarditis and heart function impairment. Our findings support previous reports linking heart disease with in utero exposure to ZIKV and provide evidence for the high prevalence and pathogenesis of cardiac disease in neonatal mice. Exposure of 2‐weeks pregnant BALB/c wild‐type (WT) mice to ZIKV can cause both nervous system disorders and heart disorders in offspring. Even if embryos (n = 17) of six pregnant BALB/c mice were surgically delivered, only 11 offspring were viable, and we could not point the exact reason why the other six foetal mice died in utero, which may be related to ZIKV exposure.

We observed ZIKV infection and functional impairment in the hearts of foetal mice (Figure 1B) and IFNα/β receptor knockout mice adult mice (Figure S1), but not in BALB/c WT adult mice hearts (Figure S1), which indicates that the hearts of immune‐deficient and foetal mice are more susceptible to ZIKV infection. Therefore, it will be appropriate to elevate the titre of ZIKV to determine whether adult WT hearts would infect because another study had reported that people who were positive for ZIKV were diagnosed with myocarditis, arrhythmias and heart failure.^6^ It will be desirable to investigate whether ZIKV infects BALB/c WT pregnant mice due to a greater burden on the existing physiological changes in organs during pregnancy.^15^

Our results using the embryo injection mouse model show important implications for explaining maternal‐infant outcome in women exposed to ZIKV during pregnancy. Thus, more attention should be paid to cardiovascular symptoms in clinical practice, and further studies to elucidate the mechanisms underlying cardiac dysfunctions associated with ZIKV infection are urgently needed.

## CONFLICT OF INTEREST

Chongzhi Bai, Jianqing Hao, Shihua Li, George Fu Gao, Yu Nie and Pengcheng Han declare that they have no conflict of interest.

## AUTHOR CONTRIBUTIONS

**Chongzhi Bai:** Data curation (lead); Formal analysis (lead); Resources (equal); Supervision (equal); Writing‐original draft (equal). **Jianqing Hao:** Investigation (equal); Methodology (equal); Resources (equal). **Shihua Li:** Data curation (equal); Resources (equal). **George Fu Gao:** Conceptualization (lead); Supervision (lead); Writing‐review & editing (lead). **Yu Nie:** Conceptualization (equal); Supervision (equal). **Pengcheng Han:** Conceptualization (lead); Supervision (lead).

## ETHICAL APPROVAL

This study was carried out in accordance with the recommendations in the Guide for the Care and Use of Laboratory Animals of the Institute of Microbiology, Chinese Academy of Sciences Ethics Committee. The protocols were approved by the Committee on the Ethics of Animal Experiments of the Chinese Academy of Sciences. Inoculations were performed under anaesthesia with ketamine hydrochloride and xylazine, and all efforts were made to minimize animal suffering. ZIKV studies were carried out under biosafety level 2 and animal BSL3 containment.

## Supporting information

Supplemental Material

## Data Availability

ZIKV strain SZ_SMGC‐1: GenBank accession number: KX266255 (https://www.ncbi.nlm.nih.gov/nuccore/KX266255.1/).
