# Supplementary material for: Myocarditis and heart function impairment occur in neonatal mice following in utero exposure to the Zika virus
Source: J Cell Mol Med. 2021 Feb 15;25(5):2730–3. doi: 10.1111/jcmm.16064 (PMC7933927; doi:10.1111/jcmm.16064)
Supplement: Supplementary file 1 — Supplemental Material [file JCMM-25-2730-s001.docx]

**Supplemental material**

**MATERIALS AND METHODS**

**Key resources table**

| REAGENT or RESOURCE | SOURCE | IDENTIFIER |
| --- | --- | --- |
| Antibodies |  |  |
| Z6 | Lab production (Unpublished data) | N/A, materials available on request from lead contact |
| Rabbit Anti- CD3 antibody | Proteintech | 17617-1-AP; AB_1939430 |
| Rabbit Anti- F4/80 antibody | Bioss | bs-11182R |
| SP Kit(Rabbit) | Bioss | SP-0023 |
| [Goat Anti-Human IgG H&L (FITC)](https://www.abcam.cn/goat-human-igg-hl-alkaline-phosphatase-ab97162.html) | Abcam | ab6854; AB_955300 |
| Anti-Sarcomeric Alpha Actinin | Abcam | ab9465; AB_2155972 |
| Rabbit two-step assay kit | ZSGB-BIO | PV-9001 |
| Critical Commercial Assays |  |  |
| Viral RNA Mini kits | Qiagen | Cat NO: 52906 |
| RNeasy Mini Kit | Qiagen | Cat NO: 74106 |
| The MB isoenzyme of creatine kinas, CK-MB | Roche | Cat NO: 11821598322 |
| Elecsys Troponin T, cTnT | Roche | Cat NO: 04491815190 |
| Experimental Models: Cell Lines |  |  |
| African green monkey: Vero | ATCC | CCL-81 |
| Baby Hamster: BHK-21 | ATCC | CCL-10 |
| Mosquito: C6/36 | ATCC | CRL-1660 |
| Human: 293T | ATCC | CRL-1573 |
| Experimental Models: Organisms/Strains |  |  |
| Virus: Zika (GenBank: KX266255) | Isolated from patient | N/A, materials available on request from lead contact |
| Mouse: BALB/c | Beijing Vital River Laboratory Animal Technology | 211 |
| Mouse: C57BL/6 | Beijing Vital River Laboratory Animal Technology | 213 |
| Mouse: *Ifnar1^-/-^* | Institute of Laboratory Animal Science, Chinese Academy of Medical Sciences | 31-11-001-C-002494 |
| **Software and Algorithms** |  |  |
| SPSS12.0.1 Package | SPSS | www.brothersoft.com/spss-268827.html |
| GraphPad Prism 5.0 | GraphPad Software | www.graphpad.com/scientific-software/prism/ |

**Viruses, ZIKV Antibody and Cells**

ZIKV strain SZ_SMGC-1 (GenBank accession number: KX266255) was isolated from a tourist who traveled back from Fiji and Samoa. Virus stocks were propagated in mycoplasma-free Vero cells and titrated by plaque forming assay. Anti-ZIKV human mAb Z6 were prepared in-house ^1^. Vero cells and BHK-21 cells were maintained in DMEM, supplemented with 10% fetal bovine serum and L-glutamine at 37 ℃ with 5% CO_2_. Mosquito C6/36 cells were maintained in RPMI 1640 medium containing 10% fetal bovine serum at 28 ℃.

**Mouse Infection Experiments**

Wild-type BALB/c (WT) mice were purchased from the Beijing Vital River Laboratory Animal Technology and IFNα/β receptor-deficient (KO) C57BL/6 mice were purchased from Institute of Laboratory Animal Science, Chinese Academy of Medical Sciences. For embryos injection, the embryos (n=17) in 6 pregnant BALB/c mice were surgically removed from 3 weeks pregnant BALB/c mice, the placenta of each embryo were inoculated with a dose of 10^4^ pfu ZIKV or Vero cells supernatants for mock group in a volume of 5 μl, then the embryos were transferred back to the uterus and sutured until their birth. Four-week-old WT and KO mice were inoculated by intraperitoneal injection (IP) with a dose of 10^4^ plaque-forming units (pfu) ZIKV in a volume of 50 μl, n = 10 for each group. Blood through the eyeball method to detect CK-MB and cTnT in serum at different time points after birth, the mice were euthanized and the hearts were collected for histological and immunofluorescence analyses.

**Echocardiography analysis**

Heart function analysis was performed using VEVO 2100 (Visual Sonics, Toronto, Canada). Anesthesia was necessary to ensure the accurate positioning of the transducer probe and to avoid movement during the measurement. Mice were continuously anaesthetized with 1.5–2% isoflurane (Pharmaceutical Partners of Canada, Richmond Hill, Canada) and warmed on a heated pad (37 °C) for long and short-axis views of heart function was obtained in B-Mode and M-Mode. Left ventricular fractional shortening (FS), ejection fraction (EF), end-systolic dimension (LVESD) and end-diastolic dimension (LVEDD) of the left ventricular were analyzed for each mouse. All data were averaged by repeating five times per mouse.

**Immunochemistry, Histology and Immunofluorescence**

For Histology, tissue was harvested and fixed overnight in 10% formalin solution, and 5-μm-thick heart sections were processed for histology by the hematoxylin and eosin HE and Masson staining.

For immunofluorescence, the frozen tissue sections (6 mm) were incubated with mouse primary monoclonal antibody anti-ZIKV (Z6; obtained in our laboratory) at 4 ℃ overnight. After washing with TBST, secondary antibody for Z6 (ab6854; Abcam), was applied for 1 hr at 37 ℃. Subsequently, the sections were washed with TBST and counterstained for DAPI (ThermoFisher Scientific).

For immunohistochemistry, the paraffin blocks of tissues were sectioned at 5 mm. Deparaffinization, rehydration and antigen retrieval were performed as described previously ^2^. The tissue sections were treated with 3% H_2_O_2_ in PBS (pH 7.6) for 10 min and blocked with normal goat serum for 10 min. The sections were then incubated at 4 ℃ overnight with primary monoclonal antibody anti-ZIKV (Z6; obtained in our laboratory) (1:200) (Key Resources Table). After rinsing with PBS, the primary antibodies were subsequently detected by incubation with biotinylated secondary antibody (Key Resources Table) followed by avidin-biotin-peroxidase (Vector Laboratories, USA). Specific binding was visualized using 3, 3’-Diaminobenzidine tetrahydrochloride. Sections were slightly counterstained with Mayer’s hematoxylin. Heart tissues from KO mice that were either uninfected or infected with ZIKV were taken for macroscopic analyses.

**ECLA**

The serum was collected after centrifugation at 800×g for 10 min. The CK-MB and cTnT were measured using ECLA kits in accordance with manufacturer’s instructions.

**RNA-Seq Analysis**

Whole hearts of mice day 9 after birth, ZIKV infected and uninfected mice (n=3) were used for global transcriptome analysis by Annoroad Co. Significantly differentially expressed genes were identified when we compared Normalized Reads Count between ZIKV infected and uninfected mice groups with p < 0.05 and Log2FoldChange > 0.263. Significance of Gene Ontology term enrichment was estimated with Fisher’s Exact Test (p value).

**Statistics**

All data were analysed with the SPSS16.0 software for Windows. The values are presented as the mean ± SEM. P-values < 0.05 were considered significant differences. Calculations were performed in Prism 6 (Graph-Pad Software).

**Ethics Statement**

This study was carried out in accordance with the recommendations in the Guide for the Care and Use of Laboratory Animals of the Institute of Microbiology, Chinese Academy of Sciences Ethics Committee. The protocols were approved by the Committee on the Ethics of Animal Experiments of Chinese Academy of Sciences. Inoculations were performed under anaesthesia with ketamine hydrochloride and xylazine, and all efforts were made to minimize animal suffering. ZIKV researches were carried out under biosafety level 2 and animal BSL3 containment.

**Reference**

1 Wang, Q. *et al.* Molecular determinants of human neutralizing antibodies isolated from a patient infected with Zika virus. *Science translational medicine* **8**, 369ra179, doi:10.1126/scitranslmed.aai8336 (2016).

2 Yu, W. *et al.* Estrogen promotes Leydig cell engulfment by macrophages in male infertility. *The Journal of clinical investigation* **124**, 2709-2721, doi:10.1172/JCI59901 (2014).


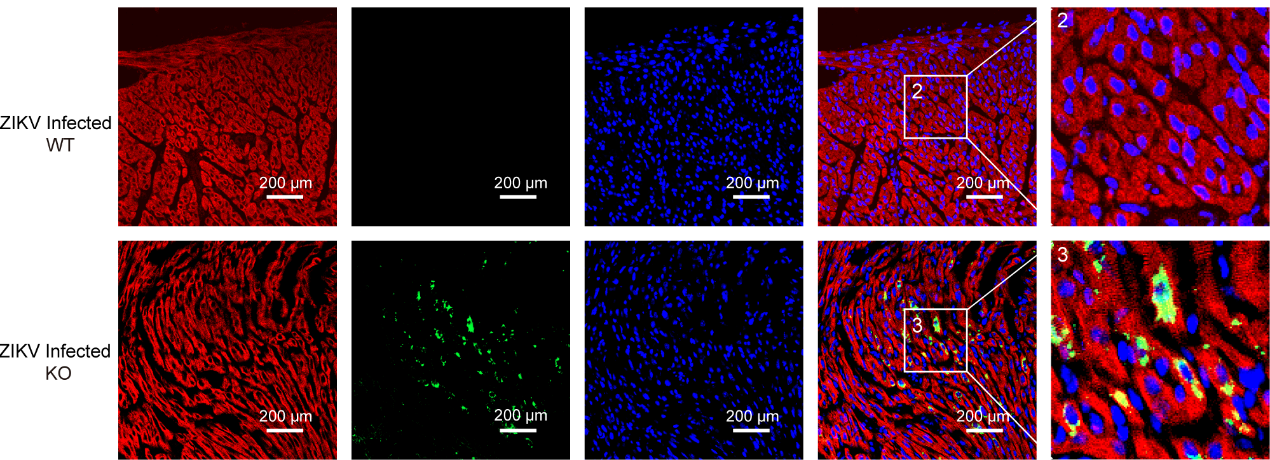


**Figure S1** ZIKV infects mouse cardiomyocyte directly in IFNα/β receptor knockout mice. Immunostaining shows that ZIKV (green) is detectable in cardiomyocytes (α-actinin^+^, red) of ZIKV-IP IFNα/β receptor knockout (KO) mice and undetectable in ZIKV-IP BALB/c wild type (WT) mice at 10 DPI.
